# Use of NAD tagSeq II to identify growth phase-dependent alterations in *E. coli* RNA NAD^+^ capping

**DOI:** 10.1073/pnas.2026183118

**Published:** 2021-03-29

**Authors:** Hailei Zhang, Huan Zhong, Xufeng Wang, Shoudong Zhang, Xiaojian Shao, Hao Hu, Zhiling Yu, Zongwei Cai, Xuemei Chen, Yiji Xia

**Affiliations:** ^a^Department of Biology, Hong Kong Baptist University, Hong Kong SAR, China;; ^b^Department of Botany and Plant Sciences, Institute of Integrative Genome Biology, University of California, Riverside, CA 92521;; ^c^State Key Laboratory of Agrobiotechnology, The Chinese University of Hong Kong, Hong Kong SAR, China;; ^d^Centre for Soybean Research, School of Life Sciences, The Chinese University of Hong Kong, Hong Kong SAR, China;; ^e^State Key Laboratory of Environmental and Biological Analysis, Department of Chemistry, Hong Kong Baptist University, Hong Kong SAR, China;; ^f^School of Chinese Medicine, Hong Kong Baptist University, Hong Kong SAR, China

**Keywords:** NAD^+^-capped RNAs, *E. coli*, gene regulation, SPAAC, NAD tagSeq II

## Abstract

Recent findings regarding nicotinamide adenine dinucleotide (NAD^+^)-capped RNAs (NAD-RNAs) indicate that prokaryotes and eukaryotes employ noncanonical RNA capping to regulate gene expression. Two methods for transcriptome-wide analysis of NAD-RNAs, NAD captureSeq and NAD tagSeq, are based on copper-catalyzed azide-alkyne cycloaddition (CuAAC) click chemistry to label NAD-RNAs. However, copper ions can fragment/degrade RNA, interfering with the analyses. Here we report development of NAD tagSeq II, which uses copper-free, strain-promoted azide-alkyne cycloaddition (SPAAC) for labeling NAD-RNAs, followed by identification of tagged RNA by single-molecule direct RNA sequencing. We used this method to compare NAD-RNA and total transcript profiles of *Escherichia coli* cells in the exponential and stationary phases. We identified hundreds of NAD-RNA species in *E. coli* and revealed genome-wide alterations of NAD-RNA profiles in the different growth phases. Although no or few NAD-RNAs were detected from some of the most highly expressed genes, the transcripts of some genes were found to be primarily NAD-RNAs. Our study suggests that NAD-RNAs play roles in linking nutrient cues with gene regulation in *E. coli*.

## Significance

Some RNAs in both prokaryotes and eukaryotes were recently found to contain the NAD^+^ cap, indicating a novel mechanism in gene regulation through noncanonical RNA capping. Copper-catalyzed azide-alkyne cycloaddition (CuAAC) click chemistry has been used to label NAD^+^-capped RNAs (NAD-RNAs) for their identification. However, copper-caused RNA fragmentation/degradation interferes with the analysis. We developed the NAD tagSeq II method for transcriptome-wide NAD-RNA analysis based on copper-free, strain-promoted azide-alkyne cycloaddition (SPAAC) click chemistry. This method was used to compare NAD-RNA and total transcriptome profiles in *Escherichia coli*. Our study reveals genome-wide alterations in *E. coli* RNA NAD^+^ capping in different growth phases and indicates that NAD-RNAs could be the primary form of transcripts from some genes under certain environments.

The 5′ end of eukaryotic messenger RNAs (mRNAs) typically contains a 7-methylguanylate (m^7^G) cap, which stabilizes the mRNA and serves as a molecular mark to recruit cap-binding proteins for RNA processing, nuclear export, and translational initiation (1–4). RNAs in prokaryotes were once thought to lack a cap and have only a 5′-triphosphorylated end from the initiation nucleotide (5). However, it has been recently reported that some RNAs in bacteria contain a nicotinamide adenine dinucleotide (NAD^+^) moiety at their 5′ end (6–10). Later, eukaryotic organisms, including yeast, mammalian cells, and *Arabidopsis* plants, were also found to produce NAD-capped RNAs (NAD-RNAs) (11–14).

A NAD-RNA can be synthesized when an RNA polymerase uses NAD^+^ as the first nucleotide, in place of adenosine triphosphate (ATP), during transcription initiation (10, 15). There is also a possibility that the NAD^+^ cap could be incorporated posttranscriptionally (11). In addition to the NAD^+^ cap, RNAs might also be capped with other noncanonical initial nucleotides (NCINs) (8, 15–20). The presence of NAD^+^ and other NCIN caps indicates another layer of gene regulation through complex RNA capping and decapping processes. In *Escherichia coli*, the NAD^+^ cap was found to enhance RNA stability (7, 15). However, molecular and physiological functions of NAD-RNAs remain elusive.

NAD captureSeq was the first method developed for transcriptome-wide identification of NAD-RNAs (7). NAD captureSeq uses ADP ribose cyclase (ADPRC)-catalyzed replacement of the nicotinamide of NAD^+^ with an alkyne followed by copper-catalyzed azide-alkyne cycloaddition (CuAAC) to label NAD-RNAs with biotin. Biotin-conjugated RNAs are then enriched by streptavidin resin to make a cDNA library that is sequenced and quantified for enrichment of tagged RNAs. Using NAD captureSeq, 53 NAD-RNAs were identified from *E. coli* (7). The method was later used for the identification of over 37 NAD-RNAs from yeast and a wider range of NAD-RNAs from mammalian cells and *Arabidopsis* plants (11–13). A recent study using NAD captureSeq identified thousands of NAD-RNAs in yeast, mostly from 5′ regions of protein-coding genes (21).

We recently developed a method termed NAD tagSeq for transcriptome-wide NAD-RNA identification and characterization in *Arabidopsis* (14, 22). Like NAD captureSeq, NAD tagSeq also uses the ADPRC-catalyzed enzymatic reaction and CuAAC click chemistry for labeling NAD-RNAs. However, in NAD tagSeq, NAD-RNAs are labeled with a synthetic RNA tag. After tagging, tagged and untagged RNAs are then identified and quantified by direct RNA sequencing using the Oxford Nanopore single-molecule sequencing technology. NAD tagSeq is simpler than NAD captureSeq, does not involve PCR amplification and cDNA library construction, and generates more detailed information on whole sequences of NAD-RNAs. Additionally, by directly sequencing both tagged and untagged RNAs without enrichment of tagged RNAs, NAD tagSeq can determine the relative abundances of NAD-RNAs and total transcripts simultaneously (14).

Both NAD captureSeq and NAD tagSeq use CuAAC click chemistry to label NAD-RNAs. However, radicals produced as a result of copper-mediated oxidation during CuAAC are detrimental to biomolecules and cause fragmentation/degradation of RNAs (23–25). RNA fragmentation during the tagging processes could cause bias toward identifying 5′-terminal fragments as NAD-RNAs and underestimation of NAD-capped transcripts. Here, we report development of a modified NAD tagging and sequencing method, termed NAD tagSeq II, which uses copper-free strain-promoted azide-alkyne cycloaddition (SPAAC) to tag NAD-RNAs. To understand possible roles of NAD-RNAs in gene regulation, we used NAD tagSeq II to simultaneously generate NAD-RNA and total transcriptome profiles in *E. coli* cells in the exponential and stationary phases. We identified at least 279 NAD-RNAs in *E. coli* in the two phases. We found that some genes produced NAD-RNAs as their major form of transcripts. Our results indicated that NAD-RNAs in *E. coli* are preferentially produced from genes that displayed differential expression in the different growth phases and might play roles in linking nutritional cues with molecular mechanisms of metabolic gene regulation.

## Results

### Development of NAD tagSeq II.

**Fig. 1.**
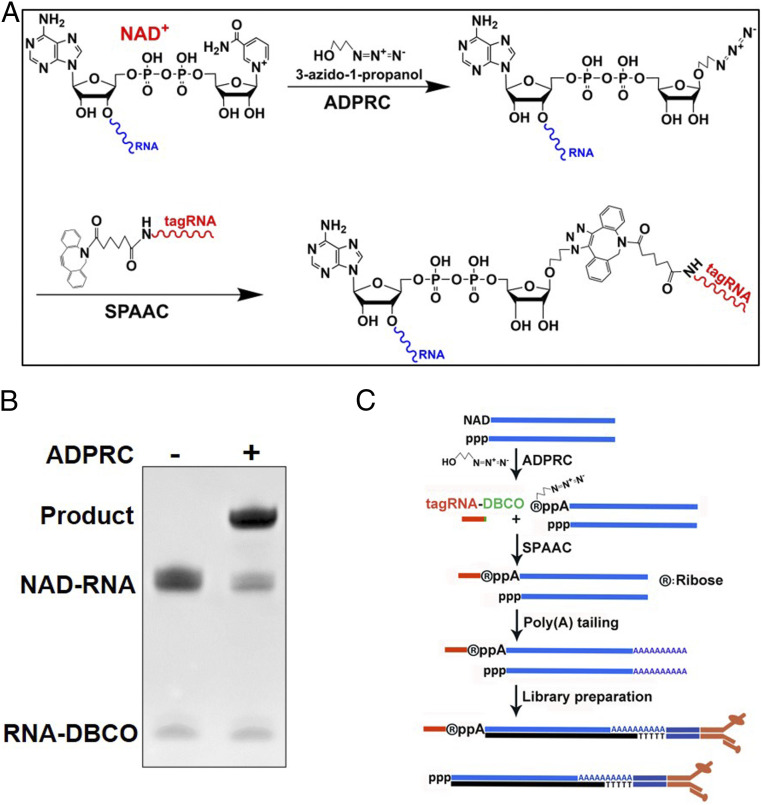
The NAD tagSeq II method. (*A*) Diagram illustrating reactions for labeling NAD-RNAs with a synthetic DBCO-modified RNA tag. In the presence of ADPRC, 3-azido-1-propanol replaces the nicotinamide of NAD-RNA. The azide-functionalized NAD-RNA molecule is then ligated to a synthetic RNA (tagRNA) with a DBCO group at its 3′ end through SPAAC. (*B*) Tagging of a 38-nt NAD-RNA with the synthetic 16-nt RNA-DBCO. The NAD-RNAs were reacted with 3-azido-1-propanol in the presence of ADPRC (ADPRC+) and then with the 16-nt RNA-DBCO, forming the ligation products. No such ligation product was observed in the reaction without ADPRC (ADPRC−). (*C*) Workflow of the NAD tagSeq II method for analysis of NAD-RNAs in *E. coli. E. coli* total RNA samples were subjected to the tagging process as described in *A*. After depletion of rRNAs from the RNA samples, the RNA samples were subjected to polyadenylation using poly(A) polymerase. The poly(A)-tailed RNA samples were used to make a library for sequencing using Oxford nanopore sequencing. A parallel experiment without ADPRC served as a control.

NAD captureSeq and NAD tagSeq label NAD-RNAs by first using ADPRC to catalyze the transglycosylation reaction that replaces the nicotinamide of the NAD cap with an alkynyl alcohol and then using CuAAC for conjugation with an azide-linked tag. To adapt SPAAC to NAD tagSeq or NAD captureSeq, an azide moiety, instead of an alkyne moiety, needs to replace the nicotinamide of NAD-RNA in the ADPRC-catalyzed reaction (Fig. 1*A*). The structural analogy between an azido alcohol and an alkynyl alcohol (the presence of the hydroxyl group) suggests that ADPRC is capable of catalyzing the transglycosylation of NAD-RNAs with azido alcohols. To test the feasibility of the reaction, we synthesized a 38-nucleotide (nt) NAD-RNA through in vitro transcription and incubated it with 3-azido-1-propanol in the presence or absence of ADPRC. The reaction product was purified and digested with nuclease P1 to release single nucleotides and either NAD^+^ or the azide-modified NAD^+^. The digest was subjected to mass spectrometry analysis. In the presence of ADPRC, a fragment (*m/z* = 643.1273) was found that differed from the NAD^+^ (*m/z* = 664.1164) observed from the reaction without ADPRC and matched the expected mass of a transglycosylation product replacing nicotinamide with 3-azido-1-propanol (*SI Appendix*, Fig. S1). The result indicates that ADPRC is indeed capable of catalyzing the transglycosylation reaction of NAD-RNA with 3-azido-1-propanol.

We compared the ADPRC-catalyzed transglycosylation reaction of the 38-nt NAD-RNA with 3-azido-1-propanol or 4-pentyn-1-ol. After the reaction, the RNA samples were purified and digested with nuclease P1. The alkyne- and azide-modified NAD^+^ fragments were quantified using mass spectrometry. It was found that 99.34% (±0.16%) and 99.41% (±0.21%) of the original NAD-RNAs were alkyne modified and azide modified in the presence of 4-pentyn-1-ol and 3-azido-1-propanol, respectively (*SI Appendix*, Fig. S2). The results showed that ADPRC could catalyze the transglycosylation of NAD-RNAs with 3-azido-1-propanol as efficiently as with 4-pentyn-1-ol.

We then used SPAAC to label the 38-nt NAD-RNA with a synthetic 16-nt RNA tag (Fig. 1 *A* and *B*). After replacing the nicotinamide of NAD-RNAs with azide through the ADPRC-catalyzed transglycosylation, the azide-functionalized NAD-RNAs were reacted with the 16-nt RNA tag linked to the DBCO (dibenzocyclooctyne) group at its 3′ end through SPAAC, resulting in conjugation of the original NAD-RNAs with the RNA tag. As shown in Fig. 1*B*, the NAD-RNA and the tag RNA were ligated in the presence of ADPRC, but such a reaction product was not observed in the absence of ADPRC, demonstrating the feasibility of SPAAC-based tagging.

**Fig. 2.**
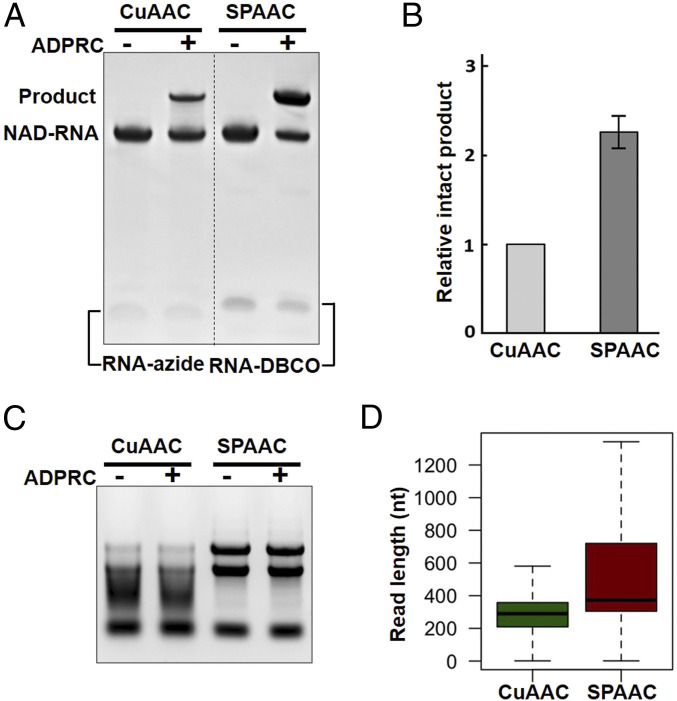
Ligation of NAD-RNAs with RNA tags through CuAAC tagging and SPAAC tagging. (*A*) Comparison of CuAAC and SPAAC tagging of a synthetic NAD-RNA. For CuAAC tagging, a 50-nt NAD-RNA was reacted with 4-pentyn-1-ol in the presence of ADPRC and then with a 16-nt RNA-azide. For SPAAC tagging, an equal amount of the NAD-RNA was reacted with 3-azido-1-propanol in the presence of ADPRC and then with a 16-nt RNA-DBCO. The amount of the 16-nt RNA-azide used in CuAAC was equal to that of the 16-nt RNA-DBCO used in SPAAC. Parallel experiments without ADPRC served as controls. The RNAs were resolved on a 15% denaturing polyacrylamide gel. (*B*) Comparison of the amount of ligation products that remained intact from the two different tagging methods. The intensity of the bands representing intact ligation products in *A* was quantified using ImageJ (https://imagej.nih.gov/ij/). Data were presented as intensity of the band from the SPAAC-based ligation relative to that of the CuAAC-based method from three independent experiments. (*C*) A representative agarose gel image of *E*. *coli* total RNA samples after being subjected to CuAAC and SPAAC tagging. RNAs became highly degraded/fragmented after CuAAC tagging. (*D*) Comparison of length of the sequencing reads obtained from NAD tagSeq (CuAAC tagging, green) and NAD tagSeq II (SPAAC tagging, purple) of *E. coli* RNA samples. Read length is shown as the number of nucleotides (nt) with the first quartile, median, and third quartile as the lower, middle, and upper lines of the boxes, respectively.

We compared SPAAC-based tagging with CuAAC-based tagging using a 50-nt synthetic NAD-RNA. The products ligated through CuAAC tagging displayed some degree of smearing (Fig. 2*A*), indicating fragmentation of RNAs, whereas SPAAC tagging resulted in over two times more intact ligation products (Fig. 2 *A* and *B*). It would be expected that fragmentation would be more severe when larger cellular RNA molecules are tagged by CuAAC. Indeed, CuAAC tagging of *E. coli* cellular RNAs showed severe RNA fragmentation as compared with SPAAC tagging (Fig. 2*C*). Moreover, overall length of sequencing reads obtained from CuAAC tagging of *E. coli* cellular RNAs was much shorter than the reads from SPAAC tagging (Fig. 2*D*; see below).

### SPAAC-Based Tagging Did Not Lead to Labeling of 5′-Tri, -Di- or -Monophosphate RNAs, FAD-RNA, 5′-Hydroxyl RNA, or Ap_4_A-RNA.

**Fig. 3.**
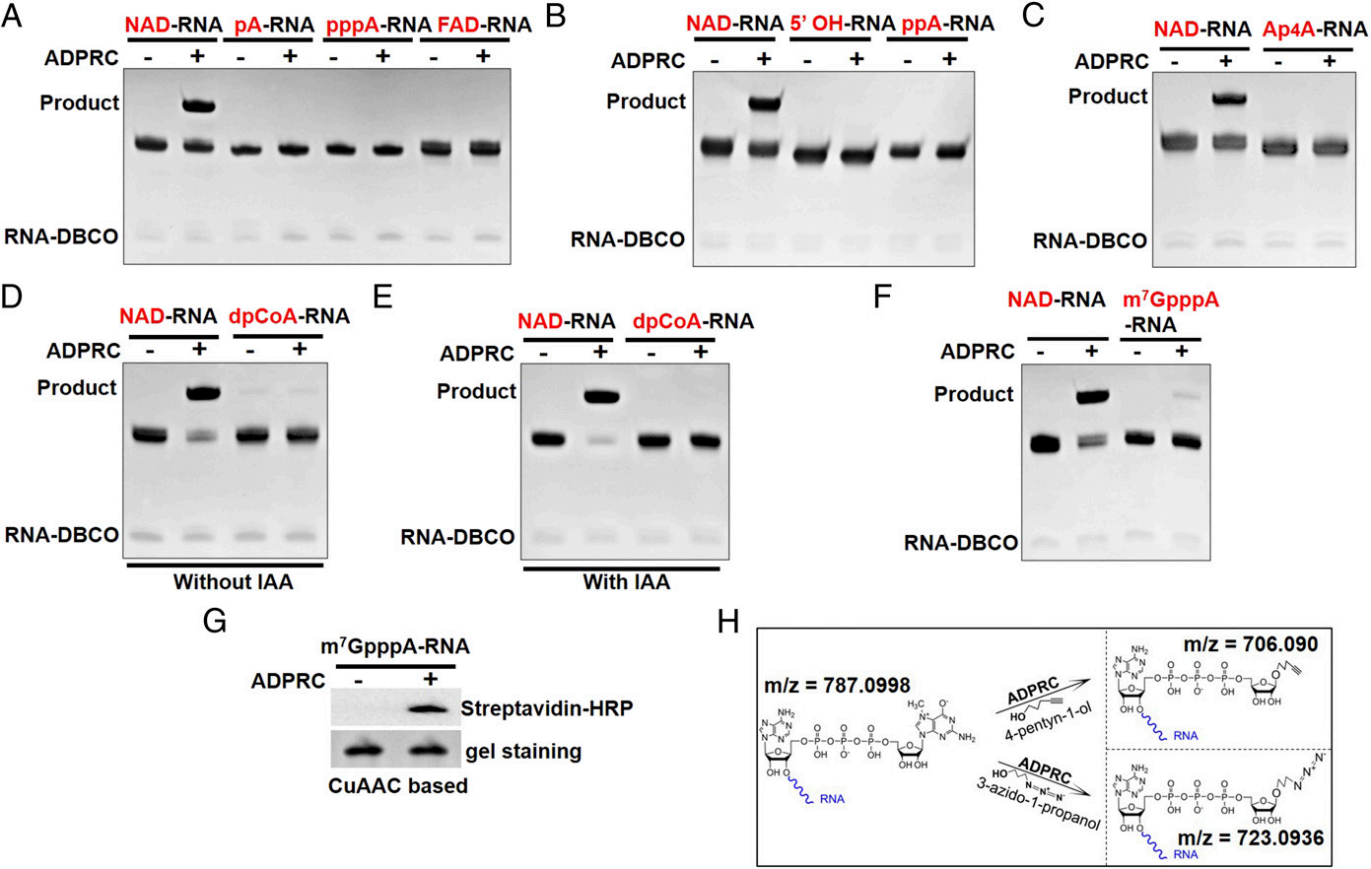
Specificity of the SPAAC-based tagging process. (*A*) Synthetic NAD-RNAs were tagged through SPAAC tagging, but 5′-monophosphate RNA (pA-RNA), 5′-triphosphate RNA (pppA-RNA), and FAD-capped RNA were not tagged with the synthetic RNA tag. (*B*) The 5′-hydroxyl RNA (5′ OH-RNA) and 5′-diphosphate RNA (ppA-RNA) were not tagged with the synthetic RNA tag. (*C*) Ap_4_A-capped RNA (Ap_4_A-RNA) was not tagged with the synthetic RNA tag. (*D*) A very small portion of synthetic dpCoA-capped RNAs was tagged through the SPAAC tagging process, irrespective of the presence of ADPRC. (*E*) The dpCoA-RNAs were first incubated with IAA and then subjected to the same tagging process as in *D*. No ligation product was detected. (*F*) A small portion of m^7^GpppA-RNAs was tagged through SPAAC tagging. (*G*) m^7^GpppA-RNAs were reacted with 4-pentyn-1-ol in the presence or absence of ADPRC and labeled with biotin through CuAAC tagging. m^7^GpppA-RNAs were found tagged with biotin in the presence of ADPRC, which were detected by Streptavidin-HRP. (*H*) Mass spectrometry analysis of alkyne- and azide-modified m^7^GpppA-RNAs after ADPRC reaction.

*E. coli* RNAs are mostly synthesized with a triphosphorylated 5′ end, but a major portion of mRNAs contains a 5′ diphosphate (26). RNA decay can result in 5′-monophosphate RNAs and 5′-hydroxyl (5′OH) RNAs (27, 28). In addition, nanoRNA-primed transcription can also produce 5′-hydroxyl RNAs in *E. coli* (29). To test whether 5′-triphosphate, 5′-diphosphate, 5′-monophosphate, or 5′-hydroxyl RNAs could be tagged through SPAAC-based tagging, we synthetized 38-nt RNAs with the above-mentioned 5′ ends by in vitro transcription and subjected them to the SPAAC-based tagging with the 16-nt RNA-DBCO tag. Unlike the 38-nt NAD-RNA, no ligation product was observed for 5′-tri, di-, or monophosphate RNA, or 5′-hydroxyl RNAs (Fig. 3 *A* and *B*).

It has recently been reported that *E. coli* RNAs can also carry dinucleoside tetraphosphates (Np_4_Ns), such as Ap_4_A, at the 5′ end (18–20). We synthetized 38-nt Ap_4_A-capped RNAs by in vitro transcription and it was not found to be tagged by SPAAC tagging (Fig. 3*C*). Other noncanonical RNA caps in prokaryotes, such as the FAD cap and the dephospho-CoA (dpCoA) cap, have also been reported (15–17). To test whether FAD-RNA or dpCoA-RNA could be tagged through SPAAC tagging, 38-nt FAD-RNA and dpCoA-RNA were synthesized and subjected to SPAAC tagging. No ligation product was observed for the 38-nt FAD-RNA (Fig. 3*A*). However, a very small portion of the 38-nt dpCoA-RNA was found to be tagged, irrespective of the presence of ADPRC (Fig. 3*D*). We assumed that the ligation might result from thiol-yne (*thiol* and an alkyne) reaction between the sulfhydryl group of the dpCoA cap and the DBCO of the RNA tag. This assumption was verified by the observation that there was no ligation product if the 38-nt dpCoA-RNA was reacted with iodoacetic acid (IAA), a sulfhydryl group blocking reagent, prior to SPAAC tagging (Fig. 3*E*). Although some cellular dpCoA-RNAs, if any, might be tagged through SPAAC tagging, the noise could be filtered out using ADPRC− samples as the negative control. If necessary, cellular RNAs can be treated with IAA prior to SPAAC tagging.

To test if the most abundant m^7^G-capped mRNAs in eukaryotic cells could be tagged by SPAAC tagging, a 38-nt synthetic m^7^GpppA-RNA (A is the first nucleotide adjacent to the m^7^G cap) was subjected to SPAAC tagging with the 16-nt RNA-DBCO tag. A small portion of the m^7^GpppA-RNA was found to be tagged (Fig. 3*F*). To determine whether CuAAC tagging could also lead to the low level labeling of m^7^G-RNA, the m^7^GpppA-RNA was subjected to ADPRC-catalyzed reaction in the presence of 4-pentyn-1-ol followed by conjugation with biotin-PEG3-azide through CuAAC. Immunoblotting analysis indeed showed that the m^7^GpppA-RNA could also be tagged with biotin (Fig. 3*G*). Mass spectrometry analysis further confirmed the presence of alkyne- and azide-modified m^7^GpppA fragments (*m/z* = 706.090 and *m/z* = 723.0936) after incubation with 4-pentyn-1-ol and 3-azido-1-propanol, respectively, in the presence of ADPRC (Fig. 3*H* and *SI Appendix*, Figs. S3 and S4). These results indicate that ADPRC can act on m^7^G-capped RNAs, although weakly. It is plausible that some NAD-RNAs in eukaryotes previously identified through the ADPRC-catalyzed labeling process might be false signals from m^7^G-mRNAs. Therefore, for using ADPRC in CuAAC- or SPAAC-based tagging to identify NAD-RNAs in eukaryotic RNA samples, an m^7^G-mRNA depletion step needs to be introduced to reduce noise from m^7^G-mRNAs before the ADPRC-catalyzed reaction is carried out, as shown by Hu et al. (30) for profiling NAD-RNAs in *Arabidopsis*.

### NAD-RNA Profiles in the Stationary Phase and Exponential Phase *E. coli* Cells.

We replaced CuAAC in the NAD tagSeq method with SPAAC-based tagging for transcriptome-wide analysis of cellular NAD-RNAs in *E. coli*. The method is named NAD tagSeq II, and the workflow is shown in Fig. 1*C*. A 39-nt synthetic RNA-DBCO was used to tag total RNA samples. The most abundant ribosomal 16S and 23S RNA were depleted after the tagging step using a bacterial rRNA depletion kit. As only poly(A)-containing RNAs can be sequenced by Oxford nanopore sequencing, an RNA polyadenylation step was carried out using yeast poly(A) polymerase. The RNA samples were then directly sequenced by Oxford nanopore RNA sequencing. Sequence reads that contained the RNA tag were deemed to be NAD-RNAs. A parallel experiment without ADPRC (ADPRC−) served as the negative control. After tagging of NAD-RNAs with the synthetic RNA tag, tagged RNAs could be enriched by hybridization to a DNA or RNA probe to increase sequencing coverage of NAD-RNAs, like in our previous analysis of *Arabidopsis* NAD-RNAs using NAD tagSeq. (14). In this study, the enrichment step was skipped so that the profiles of NAD-RNAs and total transcriptomes could be generated simultaneously and compared.

The RNA samples were extracted from *E. coli* cultures that were grown in a single batch of Luria-Bertani broth. Cells were collected after grown for 3 h (OD = 0.32; in the exponential phase) or 7 h (OD = 2.7; in the stationary phase). We used one nanopore flow cell to sequence each sample. For the initial trial to compare SPAAC and CuAAC tagging, two RNA samples from the stationary phase were tagged by CuAAC and SPAAC, respectively, and sequenced by nanopore sequencing. The overall sequence read length obtained through SPAAC tagging was much greater than that through CuAAC tagging (Fig. 2*C*). A total of 35.7% of reads from SPAAC tagging and 8.3% reads from CuAAC tagging were over 500 nt in length, respectively. A total of 611 of 1.22 million sequencing reads (0.05%) from CuAAC tagging and 4,760 of 0.97 million reads (0.49%) from SPAAC tagging were identified as NAD-RNA reads, respectively (Datasets S1 and S2). The NAD-RNA–producing genes identified by CuAAC were generally identified using SPAAC tagging, but the gene number and the NAD-RNA read member were far fewer than those identified using SPAAC (Datasets S1 and S2; see below). The result indicated that SPAAC tagging offers a much higher sensitivity and generates more accurate information on the whole sequences of NAD-RNAs than CuAAC. Further experiments were carried out by using the SPAAC-based NAD tagSeq II method. We included three biological replicates for both exponential and stationary phases, except for ADPRC− samples which had two biological replicates.

We obtained 0.49 to 1.0 million sequencing reads for each sample (*SI Appendix*, Fig. S5). Approximately 30 to 50% of the reads were rRNAs. These mapped sequencing reads are listed in Dataset S2. After removing rRNA reads, tagged reads accounted for 15,783 of the 3,412,676 reads (0.46%) in the six ADPRC+ samples. For the four ADPRC− samples, 151 of the 1,470,713 reads (0.01%) contained the tag RNA sequence. In each ADPRC− sample, generally no more than a single read from any gene was found to have the tag sequence. The exception is the ssrA transcript likely because of its very high abundance, for which 18 of its 315,450 transcripts (0.006%) contained the tag sequence. The results indicate that the noise level from this assay was very low. It also indicates that dpCoA-RNAs, if any, which might be tagged without ADPRC were extremely rare under our growth conditions and/or were not efficiently tagged in this assay.

**Fig. 4.**
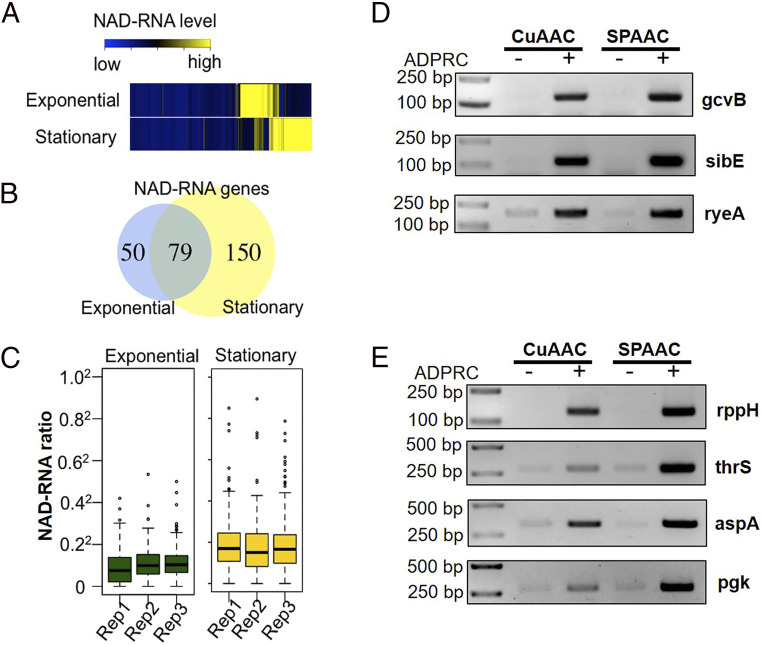
Profiles of NAD-RNAs in *E. coli* in the exponential and stationary phases. (*A*) Heatmap of normalized NAD-RNA levels in the ADPRC+ samples of stationary and exponential phases, with different colors indicating NAD-RNA levels (blue**→**yellow; low**→**high). (*B*) The numbers of genes producing high-confidence NAD-RNAs identified from the exponential and/or stationary phase cells. (*C*) Ratios of NAD-RNA reads over the total transcript reads (in square root transformation) from the high-confidence NAD-RNA–producing genes in the exponential phase (*Left*) and stationary (*Right*) phase, respectively. (*D* and *E*) Verification of NAD-RNAs by NAD capturing. RNA samples were tagged with biotin through CuAAC and SPAAC tagging. Tagged RNAs were captured by Streptavidin beads and subjected to RT-PCR analysis. Three noncoding RNAs (*D*) and four mRNAs (*E*) were selected in the analysis. For the negative control, the samples were subjected to the same treatment but without ADPRC.

Among the ADPRC+ samples, 3,988 of the 1,430,562 reads (0.28%) from the exponential phase cells and 11,795 of the 1,982,114 reads (0.6%) from the stationary phase cells were identified as NAD-RNA reads (Fig. 4*A* and Dataset S2). Although there was some overlap between the genes from which NAD-RNAs were detected in both the exponential and stationary phases, there were distinct subsets of genes that produced NAD-RNAs only in one phase (Fig. 4*A*).

The NAD-RNA–producing genes in each sample were filtered using the following criteria: 1) at least 0.2% of the transcripts from the gene were NAD-RNAs, which is about 20 times the noise level from the ADPRC− samples; and 2) there were at least two NAD-RNA reads detected from each gene. The criteria are based not on statistics but rather on our assumption that if a very small portion of transcripts from a certain gene is NAD capped, the NAD-RNAs might be produced incidentally and might not have a significant role in gene regulation. If a NAD-RNA species met the above criteria in at least two out of the three replicates, we considered it a high-confidence NAD-RNA. Using these criteria, a total of 129 genes in the exponential phase and 229 in the stationary phase were found to produce high-confidence NAD-RNAs (Fig. 4*B* and *SI Appendix*, Fig. S6 and Dataset S3). Seventy-nine of them were identified as high-confidence NAD-RNAs in both phases, bringing the total number of high-confidence NAD-RNAs to 279. On average, 5.3% of the transcripts from the 229 NAD-RNA–producing genes in the stationary phase and around 2.0% of the 129 NAD-RNA–producing genes in the exponential phase were NAD capped, respectively (Fig. 4*C*). Among these 279 NAD-RNAs, 270 were from protein-coding genes and the remaining 9 are small regulatory RNAs (Dataset S3).

The previous report using the NAD captureSeq method identified 49 NAD-RNAs from late exponential phase cells of the K-12 strain (7), the same strain used in this study. Among them, 41 were identified as NAD-RNAs in our analysis and 34 of them are in the “high-confidence” NAD-RNA category (Dataset S3). The remaining 8 NAD-RNAs were not detected, likely because the cognate genes were expressed at a very low level under our conditions. The low transcript levels for five of these eight genes could also be due to their smaller transcripts (<107 nt) which might be partially removed by the RNA Clean kit or could not be sequenced by nanopore sequencing. The smallest read detected in our analysis (not including the tag sequence) was 98 nt.

We selected seven NAD-RNAs (including three small regulatory RNAs and four mRNAs) identified in our analysis for validation through the biotin labeling/capturing approach used in NAD captureSeq. Total RNA samples were labeled with biotin through CuAAC and SPAAC tagging. Tagged RNAs were enriched using streptavidin beads and subjected to reverse transcription-PCR analysis. As shown in Fig. 4 *D* and *E*, all seven transcripts were found highly enriched by both SPAAC- and CuAAC-based biotin labeling/capturing. The result also indicates that SPAAC tagging can be adapted for NAD captureSeq, as reported by Hu et al. (30).

### The Landscapes of NAD-RNAs Varied in the Different Growth Phases.

**Fig. 5.**
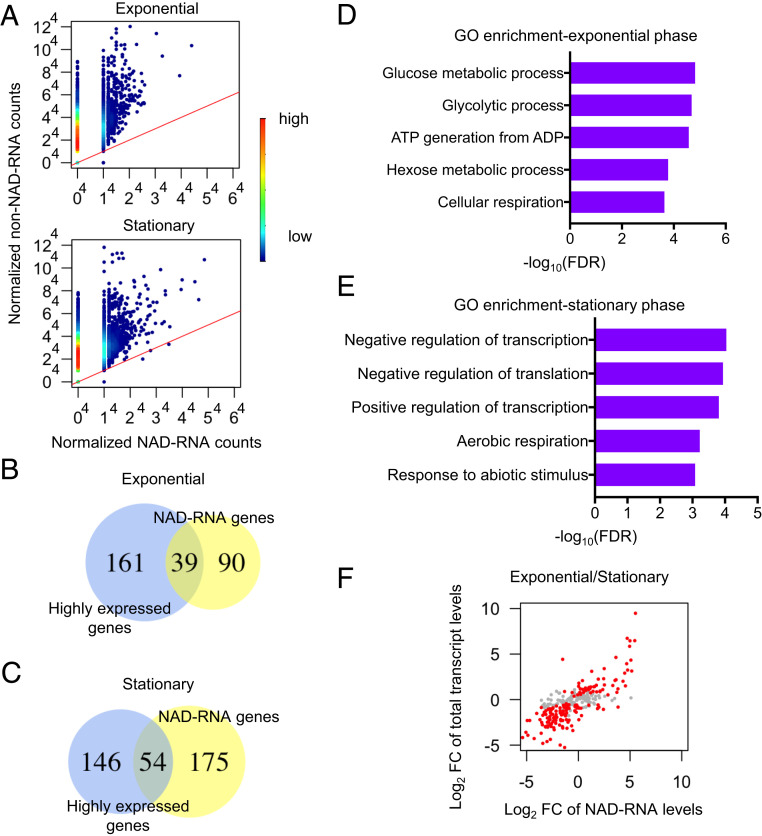
Expression profiles and functional categories of the genes producing high-confidence NAD-RNAs in the different growth phases. (*A*) Scatterplots showing normalized expression values of NAD-RNAs and non–NAD-RNA transcripts from the same genes in the exponential phase (*Left*) or stationary phase (*Right*), with colors of the dots indicating density of the points (blue→red; low→high). (*B* and *C*) Overlap between the top 200 most highly expressed genes and the high-confidence NAD-RNA–producing genes in the exponential (*B*) and stationary (*C*) phases. (*D* and *E*) Bar plots of GO enrichment analysis of the NAD-RNA–producing genes in exponential (*B*) and stationary (*C*) phases. The top five terms are shown with their FDR values in −log10 transformation. (*F*) Comparison of changes (Log2 fold change) in the NAD-RNA levels and the total transcript levels from the high-confidence NAD-RNA–producing genes between exponential and stationary phases. The values in the *x* and *y* axes represent Log2 fold differences of NAD-RNA levels and total transcript levels, respectively. The genes whose total RNA levels showed significant differential expression between the two phases are highlighted in red.

It is known that NAD^+^ capping in *E. coli* is dependent on the transcription start site (TSS) and promoter sequences (8, 15). The observation using a single promoter showed an increase in NAD^+^ capping in the stationary phase compared to the exponential phase (15). Our genome-wide NAD-RNA and total transcriptome profiles show that many highly expressed genes produced few or no NAD-capped transcripts (Fig. 5 *A*–*C* and Datasets S2 and S3). Most of the high-confidence NAD-RNAs were from moderately to lowly expressed genes (Fig. 5*A* and Dataset S3). The result further indicates that although some NAD-RNAs might be incidentally produced, most of the NAD-RNAs were produced selectively from certain genes.

In the stationary phase, the highest ratios of NAD-RNAs/total transcripts were produced from three sib genes, with 71% (sibD), 55% (sibE), and 52% (sibC) of their total transcripts detected as NAD-RNAs (Table 1). These sib genes produce small noncoding RNAs that are believed to act as antisense RNAs to transcripts of toxin-coding genes in the bacterial toxin–antitoxin system (31, 32). The result suggests that the efficiency of NAD tagSeq II for tagging and identifying NAD-RNAs might have reached over 70%, but likely not 100% yet. Therefore, the numbers were still underestimated. This result indicates that for some genes, a majority of their transcripts could be NAD^+^ capped. In the exponential phase cells, the transcripts with the highest NAD^+^ modification were from several protein-coding genes with the highest ratio of NAD-RNAs/total transcript at 38% (Table 1). The relative abundance of NAD-RNAs from many genes widely varied under the different growth phases. For instance, although over 33% of ilvL transcripts were NAD^+^ capped in the stationary phase, none of its 72 transcripts in the exponential phase were NAD-RNAs (Table 1). On the other hand, 38% of guaD transcripts in the exponential phase were NAD-RNAs, but none of its 14 transcripts in the stationary phase was found to be NAD^+^ capped. These results indicate differential production of NAD-RNAs from the same genes in response to the different growth environments.

**Table 1.** Top 10 genes that produced the highest ratio of NAD-RNAs/total transcripts in the stationary or exponential phase

		Stationary phase			Exponential phase	
Gene symbol	Product type	NAD-RNA (%)	Total counts		NAD-RNA (%)	Total counts
*sibD*	ncRNA	71.3	54		0	4
*sibE*	ncRNA	55.2	266		6.9	75
*sibC*	ncRNA	51.5	117		8.3	13
*yicG*	Protein coding	46.6	11		1.1	194
*leuA*	Protein coding	41.4	18		0	4
*yfgG*	Protein coding	39.5	34		23.7	19
*glvG*	Protein coding	33.3	5		6.7	8
*ilvL*	Protein coding	33.0	25		0	73
*yafD*	Protein coding	25.8	197		7.3	150
*ycbF*	Protein coding	23.3	9		11.1	4
*guaD*	Protein coding	0	14		38.1	9
*yfgG*	Protein coding	39.6	34		23.7	19
*amyA*	Protein coding	19.7	61		21.4	17
*Cho*	Protein coding	9.6	30		21.4	16
*ldtE*	Protein coding	10.3	64		17.9	19
*ykgS*	Protein coding	20.0	53		14.8	13
*yfcO*	Protein coding	10.1	71		13.9	52
*insG*	Protein coding	2.1	31		13.3	25
*yfjI*	Protein coding	19.2	54		12.0	15
*ybiY*	Protein coding	0	10		10.4	18

The first 10 genes produced the highest percentage of transcripts as NAD-RNAs in the stationary phase, and the last 10 genes in the exponential phase.

### The High-Confidence NAD-RNA–Producing Genes in the Different Growth Phases Are Overrepresented in Different Functional Categories.

The genes producing high-confidence NAD-RNAs in the exponential and/or stationary phases were subjected to gene ontology (GO) enrichment analysis. The 129 NAD-RNA–producing genes in the exponential phase are mostly enriched in the GO terms of metabolic and energy generation processes (Fig. 5*D* and Dataset S4). The 229 genes in the stationary phase were overrepresented in the GO terms of negative and positive regulation of transcription, negative regulation of translation and biosynthetic processes, energy production, and responses to abiotic stresses (Fig. 5*E* and Dataset S4). Many genes that produced a high percentage/level of NAD-RNAs in the stationary phase are known to be involved in response to energy depletion and other abiotic stresses, growth inhibition, and persistence/antibiotics resistance. We quantified cellular ATP and NAD^+^ levels in the exponential phase and the stationary phase. The ATP level was ∼2.5-fold lower, whereas the NAD^+^ level was slightly higher in the stationary phase than in the exponential phase (*SI Appendix*, Fig. S7).

### Most of the High-Confidence NAD-RNA–Producing Genes Were Differentially Expressed in the Different Growth Phases.

In this NAD tagSeq II assay, we did not enrich tagged RNAs so that both NAD-RNA profiles and total transcriptome profiles could be generated and compared under the different growth phases. Among all 3,907 genes whose transcripts were detected in this analysis, 946 (24%) displayed differential expression, which was defined by statistically significant difference (adjusted *P* value ≤ 0.05) in their total transcript levels between the exponential phase and stationary phase. Strikingly, among the 279 high-confidence NAD-RNA–producing genes, 208 (75%) displayed differential expression between the two phases (Fig. 5*F* and Dataset S5). The result indicates that most of the NAD-RNA–producing genes showed significant alteration in their transcript levels under the different growth phases. We then analyzed any correlation between the changes in the NAD-RNA and total transcript levels for these 279 genes under the two different growth conditions. It was found that an increase in the NAD-RNA level was generally associated with an increase in the total transcript level from the same gene (with a correlation coefficient of 0.69) (Fig. 5*F*).

### NAD-mRNAs and Non–NAD-mRNAs Have Similar Overall Coding Sequences.

**Fig. 6.**
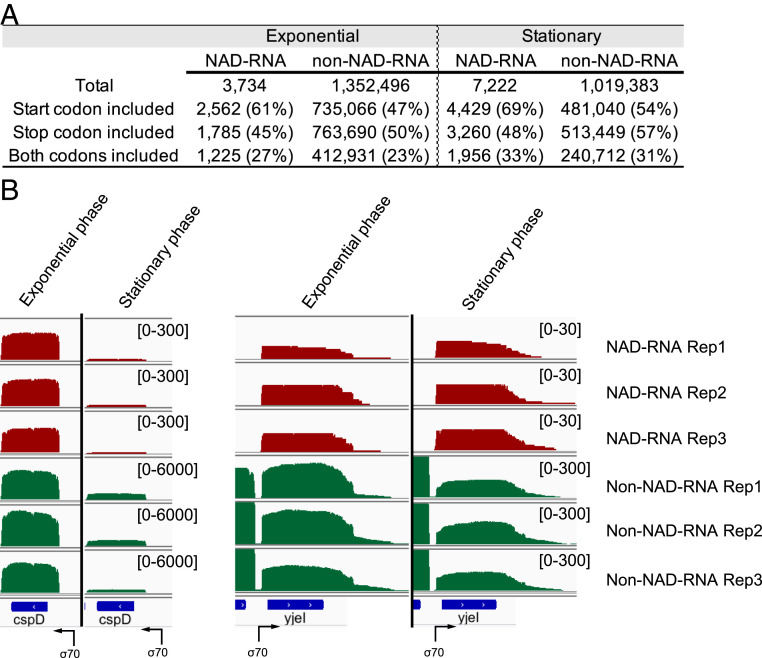
Comparison of overall sequence structures between NAD-mRNAs and non–NAD-mRNAs. (*A*) Presence of the predicted start or stop codon in NAD-mRNA or non–NAD-mRNA reads in the exponential phase and stationary phases. (*B*) Alignments of NAD-mRNA and non–NAD-mRNA reads from two protein-coding genes, cspD and yjeI. The sequencing reads from three replicates of the two growth phases were aligned using the Integrative Genomics Viewer. The number in the bracket denotes the number of reads in each sample. The coding regions of these two genes are shown at the bottom as blue bars. Black arrows indicate the transcription start sites and direction.

The coding regions of the NAD-mRNA reads and non–NAD-mRNA reads were compared to determine if those reads included the annotated start or stop codon. Overall, ∼65% of NAD-mRNAs and 50% of non–NAD-mRNAs contained the start codon sequence (Fig. 6*A*). However, the comparison of the presence of the start codon might not be very meaningful. Firstly, a dozen or so bases at the 5′ end of RNAs are often missed by the Oxford nanopore sequencing (33). Secondly, the start codon sequences in some NAD-mRNAs might not be called correctly by the regular base-calling algorithm because the presence of the nonnucleotide structure at the junction of the NAD-RNA and the RNA tag sequence formed in the ADPRC and SPAAC reactions caused incorrect calling of the first several bases downstream of the NAD^+^ cap. At the 3′ ends, ∼47% of the NAD-mRNAs and 53% of non–NAD-mRNAs contained the predicted stop codon sequence (Fig. 6*A*). The slightly lower percentage of NAD-mRNA reads with the stop codon compared to non–NAD-mRNAs might suggest that the former is slightly less stable than the latter or that synthesis of more NAD-mRNAs was prematurely terminated during transcription compared to non–NAD-mRNAs. Other than that, NAD-mRNAs and their non–NAD-mRNA counterparts generally contain similar overall sequence regions as illustrated from the alignments of the detected transcripts from two protein-coding genes (Fig. 6*B*).

## Discussion

The m^7^G cap of eukaryotic mRNAs has long been known to play essential roles in not only mRNA stability but also other steps of gene expression (1, 4, 5). The recent discoveries of NAD^+^ and other NCIN caps in RNAs of both prokaryotic and eukaryotic organisms indicate a previously unrecognized layer of gene regulation. However, molecular and physiological functions of NAD-RNAs remain elusive. It is essential to develop methods for identifying and characterizing cellular NAD-RNAs in order to understand their prevalence and functions.

The NAD captureSeq and NAD tagSeq methods used for genome-wide analysis of NAD-RNAs rely on CuAAC click chemistry for bioconjugation (7, 14). However, RNA fragmentation/degradation caused by copper during CuAAC could lead to reduced sensitivity, identification of fragmented cap-containing 5′ regions as NAD-RNAs, and loss of whole sequence information. To overcome the shortcoming, we have used copper-free SPAAC to replace CuAAC in NAD tagSeq for the development of the NAD tagSeq II method. SPAAC tagging resulted in much longer reads and a much higher efficiency in identifying NAD-RNAs than CuAAC tagging. In budding yeast, it was recently reported that most NAD-mRNAs identified using NAD captureSeq were 5′ fragments from thousands of genes, which might be incidentally produced (21). NAD-mRNAs identified from *E. coli* using NAD captureSeq also showed enrichment in 5′ fragments (7). It is possible that the enrichment of 5′ fragments of NAD-mRNAs found in those studies could partly be due to RNA fragmentation during the CuAAC reaction, leading to identification of NAD^+^ cap-containing 5′ fragments with a truncated 3′ region. Replacing CuAAC by SPAAC could also improve NAD captureSeq-based analysis of NAD-RNA profiles as shown by Hu et al. (30).

Another method termed CapZyme-Seq can provide single nucleotide resolution of 5′ ends of NCIN-capped RNAs (8). In NAD tagSeq and NAD tagSeq II, both CuAAC and SPAAC reactions create a junction between the NAD cap and the RNA tag that is not typical of a nucleotide. Such a nonnucleotide junction causes inaccurate base calling of multiple nucleotides surrounding the junction by the regular algorithm used by the Oxford nanopore system, making it difficult to determine precise 5′ ends of NAD-RNAs. In the future, machine learning algorithms could be developed for precise calling of bases surrounding the junctions to improve the NAD tagSeq II method.

NAD tagSeq and NAD tagSeq II allow simultaneous determination of the relative abundance of NAD-capped and total transcripts from the same genes, if RNA samples are directly sequenced after tagging without enrichment of tagged RNAs. Alternatively, tagged RNAs can be enriched by hybridization to a DNA probe complementary to the tag RNA sequence to increase sequencing depth of NAD-RNAs (14). In this report, we subjected RNA samples to direct sequencing without enrichment of tagged RNAs to learn the relative abundances of NAD-RNAs and total transcripts from the same genes, which could provide useful clues about the possible role of NAD-RNAs in gene regulation. Our study indicates that some *E. coli* genes could produce the majority of their transcripts as the NAD-RNA form under some conditions. In addition, some genes produced a high portion of transcripts as NAD-RNAs in the exponential phase but a low proportion in the stationary phase, and vice versa. The result indicates that production of NAD-RNAs from the same genes could be differentially regulated in response to changing environments, which is consistent with previous observations (8, 15).

The biological significance of NAD-RNAs remains elusive. A large majority of NAD-RNAs of the high-confidence NAD-RNAs identified in this study are from protein-coding genes, but some genes coding for small regulatory RNAs produced a high proportion of their transcripts as NAD-RNAs. NAD-RNAs from *Bacillus subtilis*, yeast, mammals, and *Arabidopsis* were also found to be mainly from protein-coding genes (10, 11, 13, 14, 34). It has been reported that in vitro-synthesized NAD-mRNAs were not translated in a yeast extract or in mammalian cells (11, 34). However, in *Arabidopsis*, NAD-mRNAs were found enriched in the actively translating polysomal fraction, suggesting that NAD-mRNAs could be translated (13). There is a possibility that in addition to the NAD cap, other modifications might also be present in native NAD-mRNAs and essential for translation in eukaryotic cells.

NAD^+^ and its reduced form NADH function as the most important redox cofactors in metabolism. Note that CuAAC and SPAAC reactions both act on NADH as well (30), suggesting that NAD-RNAs identified by these methods may be capped by either NAD^+^ or NADH. It is plausible that production of NAD-RNAs is influenced by NAD^+^/NADH levels, and that NAD-RNA profiles might reflect the cellular nutritional status. NAD-RNA–producing genes in the exponential phase are enriched in metabolic processes and energy production, while those in the stationary phase are enriched in negative regulation of translation (a highly energy-consuming process) and responses to abiotic stimuli. NAD-RNAs might play a role in the reciprocal connection between nutritional cues and gene expression as indicated from NAD^+^ capping of mitochondrial RNAs in eukaryotes (35).

We found that NAD-RNAs are preferentially produced from the genes that were differentially expressed in the two different growth phases. The result further suggests that NAD-RNAs are produced to regulate gene expression. In addition, we found a positive correlation in the changes in the levels of NAD-RNAs and total transcripts from the same genes. The results suggest that NAD-RNAs are produced to enhance expression of the corresponding genes, although the increasing level of NAD-RNAs might be partly due to more active transcription of the corresponding genes. NAD-RNAs could promote stability of the cognate uncapped RNAs in *E. coli* as previously observed (15). Together, our results indicate that NAD-RNAs are selectively produced to perform a specific function in gene regulation in response to changes in environmental conditions. Further studies are needed to define the precise molecular and physiological functions of NAD-RNAs.

## Materials and Methods

### General Materials.

Chemicals used in this study were purchased from Sigma–Aldrich (St. Louis, MO). DNA oligos were synthesized by Beijing Genomics Institute. RNA oligos were synthesized from Integrated DNA Technologies (IDT). All other reagents were purchased from commercial sources and used without further purification.

### In vitro transcription.

The 38-nt NAD-RNA, 38-nt pA-RNA, 38-nt pppA-RNA, 38-nt FAD-RNA, 38-nt dpCoA-RNA, 38-nt ppA-RNA, 38-nt Ap_4_A-RNA, and 50-nt NAD-RNA were produced through in vitro transcription according to the method described previously (14), with minor modifications (*SI Appendix*, *Supplementary Methods*).

### Synthetic RNAs.

RNA oligos used as the tags were synthesized at IDT. Their sequences are as follows: 5′- rGrArArCrCrUrGrArArCrCrUrGrArArC-azide-3′ for 16-nt-RNA-azide, 5′-rGrArArCrCrUrGrArArCrCrUrGrArArC-DBCO-3′ for 16-nt-RNA-DBCO, and 5′-rCrCrUrGrArArCrCrUrGrArArCrCrUrGrArArCrCrUrGrArArCrCrUrGrArArCrCrUrGrArArCrCrU-DBCO-3′ for 39-nt-RNA-DBCO.

### Iodoacetic acid treatment.

The reaction was carried out in 50 μL of solution containing 100 mM Tris⋅HCl, pH 8.0, 10 mM iodoacetic acid, 500 ng 38-nt NAD-RNAs, or dpCoA-RNAs and 50 units of murine RNase inhibitor (NEB) at 37 °C for 15 min. The RNAs were purified with RNA clean kit (Zymo) following the manufacturer’s instructions.

### Tagging of In Vitro-Transcribed RNAs.

The reaction was carried out in 50 μL of solution containing 50 mM Hepes, pH 7.0, 5 mM MgCl_2_, 200 ng RNA, 100 units of murine RNase inhibitor (NEB), 0.425 μM ADP ribosyl cyclase (Sigma), and either 10 μL 4-pentyn-1-ol (for CuAAC) or 10 μL 3-azido-1-propanol (for SPAAC) for 1 h at 37 °C. The reaction was stopped by adding 100 μL phenol/chloroform (5:1, pH 4.5) (Invitrogen). The RNA sample was purified with chloroform extraction and ethanol precipitated. The CuAAC was performed by incubating the RNA in a 50-μL reaction containing 50 mM Hepes, pH 7.0, 5 mM MgCl_2_, 15 μM 16-nt RNA-azide, 1 mM CuSO_4_, 0.5 mM THPTA, 2 mM sodium ascorbate, and 15 μM 16-nt RNA-azide at 25 °C for 30 min. The RNA was ethanol precipitated and dissolved with RNase-free H_2_O. The SPAAC was performed by incubating the RNA in a 10-μL reaction containing 155.2 mM NaCl, 2.97 mM Na_2_HPO_4_, 1.06 mM KH_2_PO_4_, pH 7.4, 10 units of murine RNase inhibitor (NEB), and 75 μM 16-nt RNA-DBCO (for tagging 50-nt NAD-RNA, 38-nt NAD-RNA, 38-nt pA-RNA, 38-nt ppA-RNA, 38-nt pppA-RNA, 38-nt 5′ OH-RNA, 38-nt FAD-RNA, 38-nt dpCoA-RNA, Ap_4_A-RNA and m^7^GpppA-RNA) for 2 h at 37 °C. An equal volume of 2× RNA loading solution (NEB) was added to the RNA samples. After incubation at 95 °C for 5 min, the RNAs were resolved on a 15% denaturing polyacrylamide gel and stained, followed by visualization using the ChemiDoc imaging system (Bio-Rad). Note that the tagging efficiency might differ under different reaction conditions, such as the concentration of tag RNAs and ADPRC and other conditions.

### Media and Culture Conditions.

*E. coli* K-12 stain MG1655 (obtained from the *E. coli* Genetic Stock Center, Yale University) was grown in Luria-Bertani (LB) broth under aeration at 37 °C. An overnight culture was diluted 1:100 into fresh medium and grown to an OD_600_ of 0.32 for the log-phase sample or to an OD_600_ of 2.7 (after 7 h of incubation) for the stationary phase sample.

### Cellular RNA Isolation.

Total RNA was extracted according to the method described by Winz et al. (23). The detailed method is described in *SI Appendix*, *Supplementary Methods*.

### Size Selection.

Size selection was conducted using the RNA Clean kit (Zymo) according to the manufacturer’s instructions. The detailed method is described in *SI Appendix*, *Supplementary Methods*.

### Tagging *E. Coli* RNA with 39-nt-RNA DBCO.

A 100-μL reaction containing 50 mM Hepes, pH 7.0, 5 mM MgCl_2_, 45 μg cellular RNA, 10 μL of 3-azido-1-propanol (Sigma), 0.85 μM ADP ribosyl cyclase (Sigma), and 100 units of murine RNase inhibitor (NEB) was incubated for 30 min at 37 °C. The reaction was stopped by adding 100 μL phenol/chloroform (5:1, pH 4.5) (Invitrogen). The mixture was centrifuged at 12,000 × *g* for 5 min at 4 °C. The upper phase was collected and mixed with 100 μL chloroform. After centrifugation, the upper phase was gently collected and mixed at a 1:3 ratio with 100% ethanol and a 0.1× volume of 3 M NaOAc (pH 5.4). The RNA sample was incubated for 30 min at −20 °C and then centrifuged at 14,000 × *g* for 20 min at 4 °C. The pellet was washed twice with 400 μL 75% ethanol, air dried, and dissolved in 10 μL RNase-free H_2_O. SPAAC was performed by incubating the RNA in a 30-μL reaction containing 155.2 mM NaCl, 2.97 mM Na_2_HPO_4_, 1.06 mM KH_2_PO_4_, pH 7.4, 30 units of murine RNase inhibitor (NEB), and 33.3 μM 39-nt-RNA-DBCO at 37 °C for 2 h. The RNA sample was mixed at a 1:3 ratio with ethanol and a 0.1× volume of 3 M NaOAc (pH 5.4). The RNA was pelleted by centrifugation at 14,000 × *g* for 20 min at 4 °C. The pellet was washed twice with 400 μL 75% ethanol, air dried, and dissolved in 40 μL RNase-free H_2_O.

### Biotin Labeling and Immunoblotting Analysis.

The 200 ng of 38-nt m^7^GpppA-capped RNA was subjected to ADPRC reaction using 4-pentyn-1-ol as substrate, following the procedure for tagging of in vitro transcribed RNAs mentioned above. Then CuAAC-based biotin labeling was performed following the procedure as described (22). Unreacted biotin-PEG3-azide were removed by adding 100 μL phenol/chloroform (5:1, pH 4.5) (Invitrogen). The mixture was centrifuged at 12,000 × *g* for 5 min at 4 °C. The upper phase was collected and mixed with 100 μL chloroform. After centrifugation, the upper phase was gently collected and mixed at a 1:3 ratio with 100% ethanol and a 0.1× volume of 3 M NaOAc (pH 5.4). The RNA sample was incubated for 30 min at −20 °C and then subjected to centrifugation at 14,000 × *g* for 20 min at 4 °C. The RNA sample was separated on a 10% denaturing polyacrylamide gel electrophoresis gel and transferred to a positively charged nylon membrane Hybond-N (Amersham Pharmacia Biotech) and probed with horseradish peroxidase (HRP)-conjugated Streptavidin (Thermo Scientific). The signal was detected using ECL Western Blotting Substrate (Pierce) and blot image was acquired using Chemidoc XRS+ Imager (Bio-Rad).

### Ribosomal RNA Removal.

Removal of 16S and 23S rRNAs was performed using MICROBExpress kit (Invitrogen) which includes binding solution, capture oligonucleotides, and derivatized magnetic beads (see *SI Appendix*, *Supplementary Methods* for details).

### poly(A) Tailing.

A 60-μL reaction containing 20 mM Tris⋅HCl, pH 7.0, 600 μM MnCl_2_, 20 μM ethylenediaminetetraacetic acid (EDTA), 200 μM dithiothreitol, 6 µg acetylated bovine serum albumin (BSA), 10% glycerol, 4 μg RNA, 1 mM ATP, 60 units of murine RNase inhibitor (NEB), and 100 units of yeast poly(A) polymerase (MCLAB) was incubated for 20 min at 37 °C. The reaction was stopped by adding RNA binding solution and the RNA sample was purified with RNA Clean kit (RCC-5) (Zymo) following the manufacturer’s instructions.

### Library Preparation and Sequencing.

For each RNA sample, 1 μg poly(A)-tailed RNA was used to prepare a library using the Nanopore Direct RNA Sequencing Kit (RNA-0002) following the manufacturer’s instructions (Oxford Nanopore Technologies). Each library was loaded onto a flow cell (R9.4) and sequenced on the sequencing device GridION. Base calling was conducted using Guppy software (Oxford Nanopore Technologies).

### NAD Capture RT-PCR.

A total of 100 μg total RNA and IAA-treated 100 μg total RNA were subjected to ADPRC reaction using 4-pentyn-1-ol and 3-azido-propanol as substrate, respectively, following the procedure as tagging of in vitro-transcribed RNAs. Then CuAAC-based biotin labeling was performed following the procedure as described (7). SPAAC-based biotin labeling was performed in a 30-μL reaction containing 155.2 mM NaCl, 2.97 mM Na_2_HPO_4_, 1.06 mM KH_2_PO_4_, pH 7.4, 30 units of murine RNase inhibitor (NEB), and 3.3 mM biotin-PEG4-DBCO (Sigma) at 37 °C for 1 h. Unreacted biotin-PEG4-DBCO and biotin-PEG3-azide were removed by adding 100 μL phenol/chloroform (5:1, pH 4.5) (Invitrogen). The mixture was centrifuged at 12,000 × *g* for 5 min at 4 °C. The upper phase was collected and mixed with 100 μL chloroform. After centrifugation, the upper phase was gently collected and mixed at a 1:3 ratio with 100% ethanol and a 0.1× volume of 3 M NaOAc (pH 5.4). The RNA sample was incubated for 30 min at −20 °C and then centrifuged at 14,000 × *g* for 20 min at 4 °C. The pellet was washed twice with 400 μL 75% ethanol, air dried, and dissolved in 300 μL buffer containing 50 mM Tris⋅HCl, 0.5 M LiCl, 1 mM EDTA, pH 7.5. The RNA solution was incubated with 500 μg magnetic Streptavidin C1 beads (Invitrogen) 37 °C for 30 min and the beads were washed with RNase-free water. The RNAs were eluted by incubating the beads in 80 μL elution buffer containing 50 mM Tris⋅HCl, pH 7.5, 95% formamide, 10 mM EDTA, and 2 mM biotin at 65 °C for 5 min. The RNA was ethanol precipitated and reversed transcribed using PrimeScript RT Master Mix (Takara). PCR was performed using PCR primers (*SI Appendix*, Table S2).

### Preprocessing and Analysis of Sequencing Reads.

The bioinformatic analysis was conducted using our home-made pipeline, TagSeqTools (36). Tagged and nontagged reads were identified using the TagSeek module with default setting (similarity = 12). Two sets of reads generated from the TagSeek step were subjected to TagSeqQuant for aligning to both the reference genome and transcriptome of *E. coli* strain K-12 substrain MG1655 (37), with the default alignment parameters of minimap2 (38). In this step, gene coverage files of tagged and nontagged reads were generated for visualization, and raw counts of genes were produced for further analysis.

### Correlation, Functional Analysis, and Visualization.

Pairwise Pearson correlation values were calculated using the statistics package in R 3.5.1 (http://www.r-project.org/). GO enrichment analysis was performed using the binomial test in the PANTHER database (39), and terms with a *P* value of <0.05 after false discovery rate correction were considered to be significant. The Integrative Genomics Viewer (IGV) (40) was used to visualize NAD-RNAs and non–NAD-RNAs.

### Correlation of NAD-RNA Levels with Total Transcript Levels.

The DESeq2 package (41) was adopted to calculate the difference in NAD-RNA levels and total transcript levels between stationary and exponential phases. The raw counts were scaled by the library sizes using the default normalization settings in DESeq2, with a significance test for differential expression based on the negative binomial distribution.

## Supplementary Material

Supplementary File

Supplementary File

Supplementary File

Supplementary File

Supplementary File

Supplementary File

## Data Availability

Raw fastq data have been submitted to the National Center for Biotechnology Information Gene Expression Omnibus repository (accession no. GSE153253). Additional experimental procedures are included in *SI Appendix*, *Supplementary Methods*.
