# Supplementary material for: Use of NAD tagSeq II to identify growth phase-dependent alterations in E. coli RNA NAD+ capping
Source: Proc Natl Acad Sci U S A. 2021 Mar 29;118(14):e2026183118. doi: 10.1073/pnas.2026183118 (PMC8040648; doi:10.1073/pnas.2026183118)
Supplement: Supplementary File [file pnas.2026183118.sapp.pdf]

SUPPLEMENTARY INFORMATION

Supplementary Fig 1. Mass spectrometry analysis of the transglycosylation on NAD-RNA catalyzed by ADPRC in the presence of 3-azido-1-propanol.

Supplementary Fig 2. Comparison of ADPRC efficiency on the 38-nt NAD-RNAs in the presence of 4-pentyn-1-ol and 3-azido-1-propanol.

Supplementary Fig 3. Mass spectrometry analysis of transglycosylation on m^7GpppA-RNA catalyzed by ADPRC in the presence of 3-azido-1-propanol.

Supplementary Fig 4. Mass spectrometry analysis of transglycosylation on m^7GpppA-RNA catalyzed by ADPRC in the presence of 4-pentyn-1-ol.

Supplementary Fig 5. Correlation of the abundances of NAD-RNAs in three replicates of the stationary and exponential phase cells.

Supplementary Fig 6. Venn plot of high confidence NAD-RNAs from the samples of two growth phases.

Supplementary Fig 7. LC-MS analysis of cellular ATP and NAD^+ levels in exponential and stationary phases.

Supplementary Table 1. Numbers of sequencing reads from all samples.

Supplementary Table 2. List of the primers used in reverse transcription-PCR of NAD captured RNA samples.

Supplementary methods

The following datasets are provided as separate files:

Dataset S1. List of all sequencing reads from CuAAC tagging (separate file)

Dataset S2. List of all sequencing reads from SPAAC tagging (separate file)

Dataset S3. List of the high-confidence NAD-RNAs in the exponential phase and the stationary phase (separate file)

Dataset S4. GO enrichment analysis of genes producing high-confidence NAD-RNAs (separate file)

Dataset S5. Differences in the levels of NAD-RNAs and total transcripts from the genes producing high-confidence NAD-RNAs between the two growth phases (separate file)

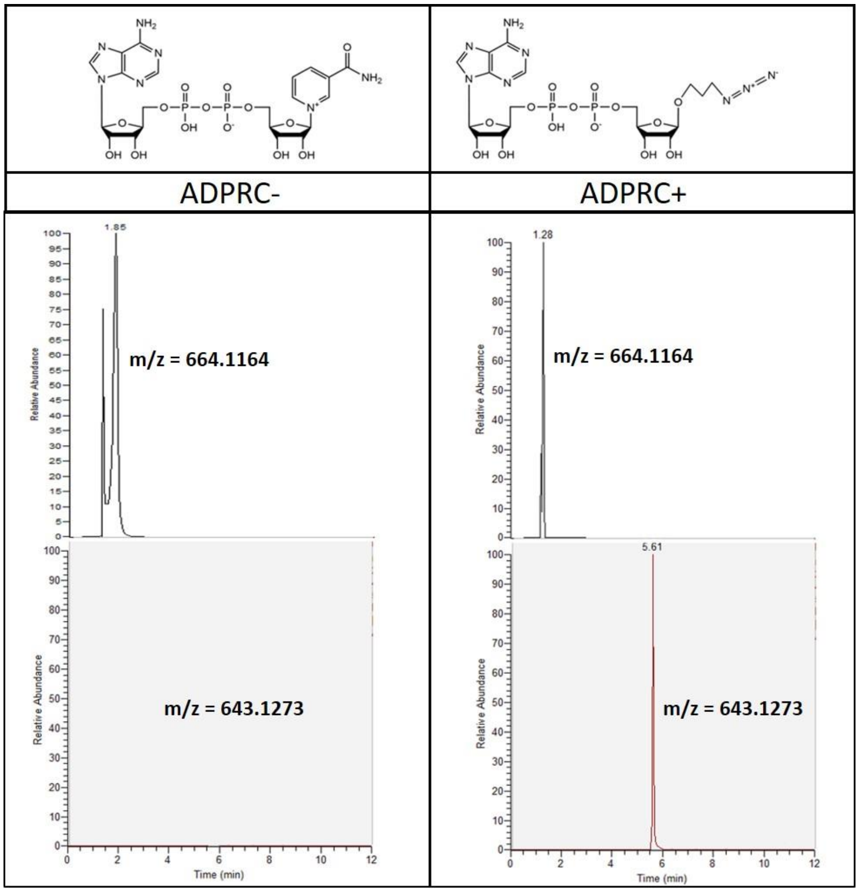

Supplementary Fig 1. Mass spectrometry analysis of transglycosylation product of NAD-RNA with 3-azido-1-propanol in the absence (ADPRC-) or presence of ADPRC (ADPRC+). The 38-nt NAD-RNA was reacted with 3-azido-1-propanol in the absence or presence of ADPRC. The RNA samples were purified and then digested with nuclease P1. The digests were applied to LC-MS system coupled with Q-Exactive ESI-Q-Orbitrap Mass Spectrometer in the positive ion mode. HPLC-MS extracted ion chromatograms of the peak $[M]^+$ m/z=664.1164 of free NAD^+ (left panel) and appearance of a peak at m/z=643.1273 (elution time 5.5-6.0 min) upon ADPRC reaction (right panel).

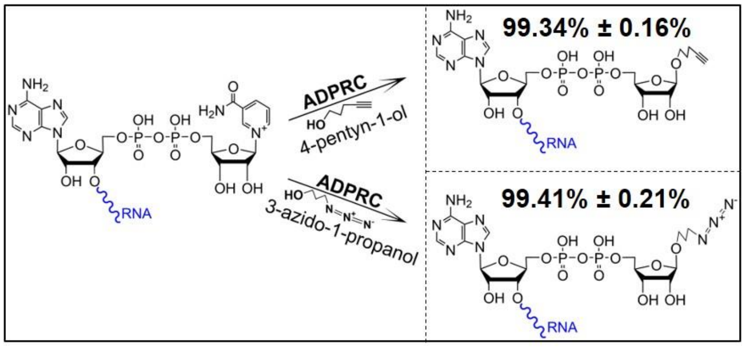

Supplementary Fig 2. Comparison of ADPRC efficiency on the 38-nt NAD-RNAs in the presence of 4-pentyn-1-ol and 3-azido-1-propanol. Data were presented as the amount of alkyne- or azide-modified NAD-RNAs in the presence of ADPRC relative to the ADPRC- control from three independent experiments.

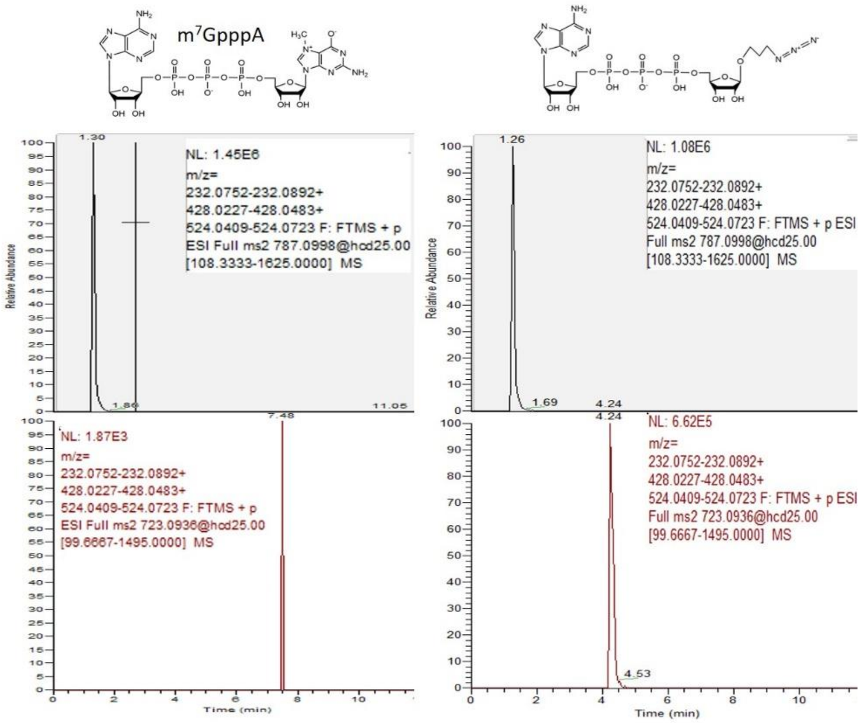

Supplementary Fig 3. Mass spectrometry analysis of transglycosylation product of m7GpppA-RNA with 3-azido-1-propanol in the absence or presence of ADPRC. A 38-nt m^7GpppA-RNA was reacted with 3-azido-1-propanol in the absence or presence of ADPRC. The RNA samples were purified and then digested with nuclease P1. The digests were applied to LC-MS system coupled with Q-Exactive ESI-Q-Orbitrap Mass Spectrometer in the positive ion mode.

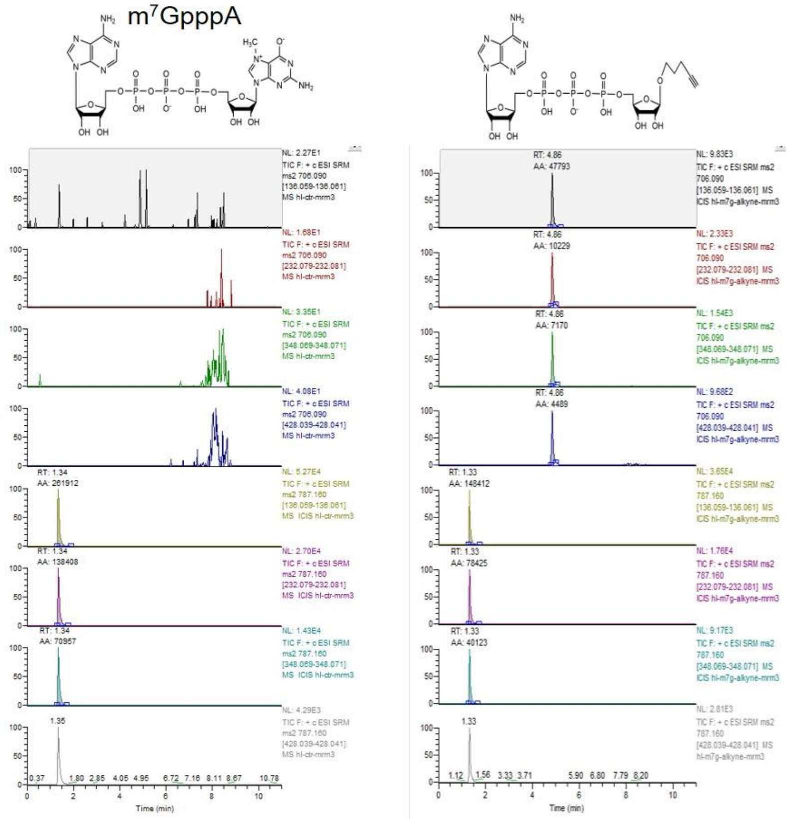

Supplementary Fig 4. Mass spectrometry analysis of the transglycosylation product of m7GpppA-RNA with 4-pentyn-1-ol in the absence or presence of ADPRC. The 38-nt m^7GpppA-RNA was reacted with 4-pentyn-1-ol in the absence or presence of ADPRC. The RNA samples were purified and then digested with nuclease P1. The digests were applied to LC-MS coupled with Q-Exactive ESI-Q-Orbitrap Mass Spectrometer in the positive ion mode.

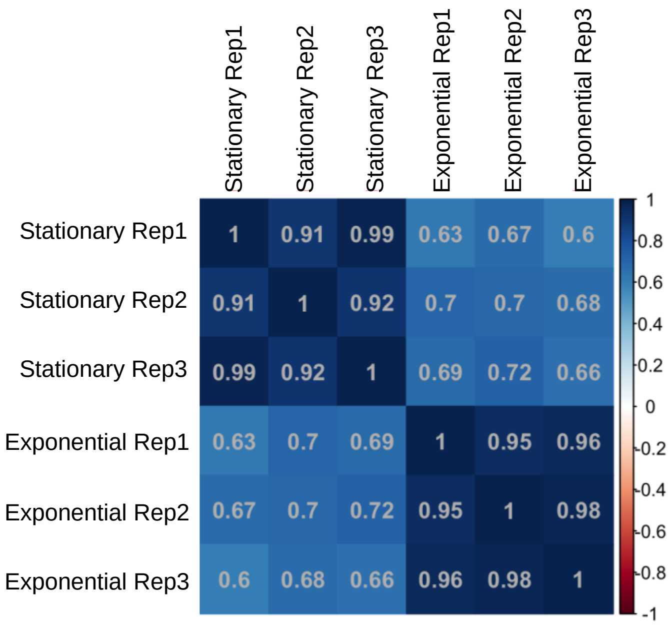

Supplementary Fig 5. Correlation of the abundances of NAD-RNAs in three replicates of the stationary and exponential phase cells. Pairwise Pearson correlations are shown for each replicate in the experiments. The darker the color is, the higher the correlation between the paired samples.

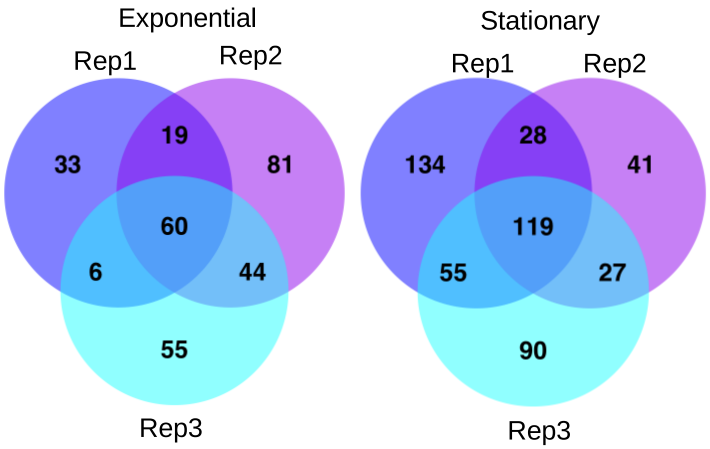

Supplementary Fig 6. Venn plot of high confidence NAD-RNAs from the samples of two growth phases. The numbers of genes producing high confidence NAD-RNAs in three replicates of the exponential phase (left) or stationary phase (right). Genes shared among a dataset are shown as overlapping.

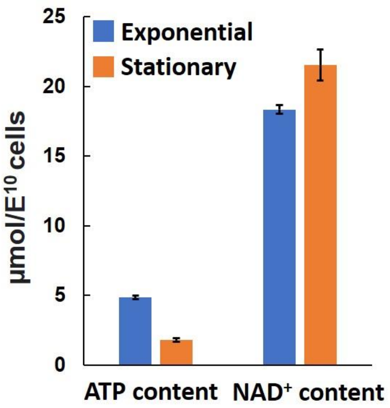

Supplementary Fig 7. LC-MS analysis of cellular ATP and NAD^+ levels in exponential and stationary phases. Metabolites were extracted from cells at a time point in exponential phase and stationary phase, respectively. Data were presented as amount of analyte normalized to cell number from five replicates.

Supplementary Table 1: Numbers of sequencing reads from all samples

Sample	Total reads	Tagged reads	Mapping ratio of tagged reads
Exponential Rep1 (+)	868,511	1,093	93.34%
Exponential Rep2 (+)	1,009,435	1,867	94.47%
Exponential Rep3 (+)	862,449	1,437	94.14%
Exponential Rep1 (-)	486,603	91	97.22%
Exponential Rep2 (-)	619,624	127	98.28%
Stationary Rep1 (+)	971,048	4,760	74.19%
Stationary Rep2 (+)	1,005,449	3,230	76.94%
Stationary Rep3 (+)	1,026,364	4,386	73.97%
Stationary Rep1 (-)	749,974	92	98.05%
Stationary Rep2 (-)	727,722	140	95.85%

Supplementary Table 2: List of primers used in NAD capture RT-PCR

primer	5'-3' sequence
thrS_FP	CGTTAATGGCGAACTGGTTGATG
thrS_RP	CGTCGTAGTTTTTCTCAGCAAGC
pgk_FP	CGTATCCGTGCTTCTCTGCC
pgk_RP	TTTGGACAGGGTTTCGTCGTC
aspA_FP	CAGTGATATTCCTGAATTTGTTCGCG
aspA_RP	GTCGGGTAGGCGTCGTTAGTG
sibE_FP	GGGAGGATTTCTCCCCCCTC
sibE_RP	GAAGGGGCCTTGTATAAGGAAAGG
ryeA_FP	GAGACCGAACACGATTCCTGTATTC
ryeA_RP	CAGCTGATGACCACCACG
gcvB_FP	TATCGGAATGCGTGTTCTGGTG
gcvB_RP	AGACAGGGTAAATGTACAGGAAGTG
rppH_FP	ATTTGGTCAGCACTCCTGGC
rppH_RP	AGTTACGCGTTGAAGCAAGGAT

Supplementary methods

Mass spectrometry analysis of the tagging product of NAD-RNA or m7GpppA-RNAs. A reaction (50 µL) containing 50 mM HEPES, pH 7.0, 5 mM $MgCl_2$, 500 ng 38-nt NAD-RNA (or m^7GpppA-RNA), 10 µL 3-azido-1-propanol (or 4-pentyn-1-ol) (Sigma), 0.425 µM ADP-ribosyl cyclase (Sigma) was carried out at 37°C for 30 min. The reaction without ADP-ribosyl cyclase was used as control. The reaction was stopped with phenol/chloroform extraction, and the RNA was ethanol precipitated. The RNA was incubated with 1 unit of nuclease P1 in a 50-µl reaction including 300 mM NaOAc, pH 5.4, and 2 mM $ZnCl_2$ for 30 min at 37°C. The digest was applied to an Ultimate 3000 UHPLC (Thermo Fisher Scientific) LC system coupled with a Q-Exactive ESI-Q-Orbitrap Mass Spectrometer (Thermo Fisher Scientific) in positive mode. For chromatographic separation, an Aquity UPLC BEH C18 column (1.7 µm particle size, 2.1 mm i.d., 100 mm length, Waters) was used. For each sample, 10 µL of the re-dissolved solution was injected. Mobile phase A was 0.1% aqueous NH_4OAc, and mobile phase B was 100% methanol. The flow rate was a constant 0.3 mL/min, and the mobile phase composition was set as follows: 2% B for 1 min; linear increase over 3 min to 15% B; second linear increase over 2 min to 100% B, which was maintained for 1 min before returning linearly to 2% B over 0.5 min. For MS analysis, electrospray ionization (ESI) was used with spray voltage of 3.5 kV. The auxiliary gas flow rate was 10 arbitrary units and sheath gas flow rate was 35 psi. The capillary temperature was 350°C. The mass resolution was set as 70,000, the scan range was m/z 75 – 800, and the S-lens RF level was 55. The collision voltage was set as 30.0 V for MS/MS analysis.

In vitro transcription for synthesis of RNAs with different caps

38-nt NAD-RNA, 38-nt pA-RNA, 38-nt ppA-RNA, 38-nt pppA-RNA, 38-nt FAD-RNA, 38-nt dpCoA-RNA, Ap_4A-RNA and 50-nt NAD-RNA were produced through in vitro transcription according to the method described previously (1), with minor modifications. Transcription was performed in 400-µL reactions containing 40 mM Tris-HCl, pH 8.0, 1 mM spermidine, 22 mM $MgCl_2$, 0.01% Triton-X-100, 5mM DTT, 5% DMSO, 1 mM GTP, 1 mM CTP, 1 mM UTP, 1 µM double-stranded DNA template, 400 units of murine RNase inhibitor (NEB), 10 µg T7 RNA polymerase and either 1 mM NAD (for 38-nt NAD-RNA and 50-nt NAD-RNA), 1 mM AMP (for 38-nt pA-RNA), 1 mM ADP (for 38-nt ppA-RNA), 1 mM ATP (for 38nt pppA-RNA), 4 mM FAD (for 38-nt FAD-RNA), 4 mM dephospho-CoA (for 38-nt dpCoA-RNA), 4 mM Ap_4A (Sigma, for 38-nt Ap_4A-RNA), or 4 mM m^7GpppA (NEB, for 38-nt m^7GpppA-RNA). The reaction was incubated at 37 °C for 6 h. Template for 38-nt pppA-RNA was prepared by annealing the DNA oligo 5’-GCTAATACGACTCACTATTAACAGTGTTCTTGTTGCTGTAGAACACAAACAGAACAT-3’ with 5’-ATGTTCTGTTTGTGTTCTACAGCAACAAGAACACTGTTAATAGTGAGTCGTATTAGC-3’. Template for 50-nt NAD-RNA was prepared by annealing the DNA oligo 5’-GATCACTAATACGACTCACTATTACGTGGCTCTGCTCGTGTGGCTCTGCTCGTGTCGCTCTGTTGTCGCGCGT-3’ with 5’-ACGCGCGACAACAGAGCGACACGAGCAGAGCCACACGAGCAGAGCCACGTAATAGTGAGTCGTATTAGTGATC-3’. Templates for producing RNAs with other caps are different from that for pppA-RNA. Templates for 38-nt NAD-RNA, 38-nt pA-RNA, 38-nt FAD-RNA, 38-nt dpCoA-RNA, 38-nt Ap_4A-RNA, and 38-nt ADP-RNA were prepared according to the previously described method (1). The transcription products were incubated with 20 units of DNase I (NEB)

for 20 min at 37 °C. The RNA was separated by 10% urea polyacrylamide gel electrophoresis and stained by RedSafe (iNtRON). Gel slices containing the RNAs were excised and crushed. The RNAs were eluted from the gel slice, ethanol precipitated and purified with RNA Clean kit (Zymo) following the manufacturer's instructions. 5'-hydroxyl (5'-OH) 38-nt RNA was prepared by dephosphorylation of the pA-38-nt RNA. The reaction was performed in 40-μL reaction containing 1 ug of pA-38-nt RNA and 4 units of Calf-intestinal alkaline phosphatase (Invitrogen) in 50 mM Tris-HCl, pH 8.5, 100 μM EDTA for 20 min at 37 °C and purified with with RNA Clean kit (Zymo).

Cellular RNA isolation. Cell pellets were collected by centrifugation at 5,000 g for 5 min at 25°C, and then thoroughly re-suspended in 10 ml TES buffer (10 mM Tris-HCl, pH7.5, 10 mM EDTA and 0.5% (w/v) SDS). The cells were mixed with 10 ml phenol/chloroform (5:1, pH 4.5) (Invitrogen) and immediately lysed by incubating for 5 min at 65°C with vigorous rotation. The sample was then vortexed for 3 min at 25°C before centrifugation at 12,000 g for 10 min at 4°C. The upper phase was collected and mixed with 3 ml of phenol/chloroform (5:1, pH4.5) before centrifugation at 12,000 g for 10 min at 4°C. The upper phase was mixed with 3 mL of chloroform. After centrifugation at 12,000 g for 10 min at 4°C, the upper phase was gently collected and mixed at a 1:1 ratio with isopropanol and a 0.1x vol. of 3 M NaOAc (pH 5.4). The mixture was incubated for 10 min at 25°C. RNA was pelleted by centrifugation at 14,000 g for 20 min at 4°C. The pellet was washed twice with 1 mL 75% ethanol, air dried, and dissolved in RNase-free H_2O.

Tagging *E. coli* RNA with 41-nt-RNA azide. A 100 μL reaction containing 50 mM HEPES, pH 7.0, 5 mM $MgCl_2$, 45 μg cellular RNA, 10 μl of 4-pentyn-1-ol (Sigma), 0.85 μM ADP-ribosyl cyclase (Sigma) and 100 units of murine RNase inhibitor (NEB) was incubated for 30 min at 37°C. The reaction was stopped by adding 100 μL phenol/chloroform (5:1, pH 4.5) (Invitrogen). The mixture was centrifuged at 12,000 g for 5 min at 4°C. The upper phase was collected and mixed with 100 μL chloroform. After centrifugation, the upper phase was gently collected and mixed at a 1:3 ratio with 100% ethanol and a 0.1x vol. of 3 M NaOAc (pH 5.4). The copper-catalyzed azide-alkyne cycloaddition (CuAAC) was conducted by incubating the RNA in 100 μL reaction containing 50 mM HEPES (pH7.0), 5 mM MgCl2, 10 μM 41-nt-RNA-azide (1), 1 mM CuSO4, 0.5 mM THPTA and 2 mM Sodium Ascorbate at 24°C for 30 min with gentle vortexing. The RNAs were then purified with RNA clean kit (Zymo).

Ribosomal RNA removal. Removal of 16S and 23S rRNAs was performed using MICROBExpress kit (Invitrogen) which includes binding solution, capture oligonucleotides and derivatized magnetic beads. 40 μL RNAs were mixed with 900 μL binding solution and 18 μL capture oligonucleotides. The mixture was incubated for 10 min at 70°C to denature the secondary structures of RNAs, and then incubated for 15 min at 37°C to allow the capture oligonucleotides to hybridize to 23S and 16S rRNAs. Then, 250 μL magnetic beads were added to the RNA-capture oligonucleotides mixture. The solution was incubated for 15 min at 37°C. The rRNA-capture oligonucleotide-magnetic beads complex was pulled down using a magnetic stand (NEB) and the supernatant was transferred to a new tube. The rRNA-depleted RNA fraction was concentrated using RNA Clean kit (Zymo). Then a size selection was conducted with the purpose of removing 5S, tRNAs and excess tagRNA following the method described above.

RNA size selection. Size selection of RNAs was performed using RNA Clean kit (Zymo) according to the manufacturer's instructions. Cellular RNA (50 µg) was diluted with RNase-free H_2O to a 200 µL solution. The RNA sample was mixed at a 1:2 ratio with RNA binding buffer/ethanol (1:1) and then loaded on a spin column followed by centrifugation at 12,000 g for 30 s at 4°C. 400 µL RNA preparation solution was added to the column and centrifuged at 12,000 g for 30 s at 4°C. The column was then washed by adding 700 µL washing buffer before centrifugation. The column was washed again with 400 µL washing solution followed by centrifugation. The column was dried by centrifugation at 12,000 g for 1 min at 4°C. RNase-free H_2O (50 µL) was added to the column to elute the RNAs. The eluted RNAs were collected by centrifugation at 12,000 g for 2 min at 4°C.

Tagging of *E. coli* total RNA for agarose gel electrophoresis analysis. The reaction was carried out in 100 µL of solution containing 50 mM HEPES, pH7.0, 5 mM $MgCl_2$, 20 µg RNA, 100 units of murine RNase inhibitor (NEB), 0.425 µM ADP-ribosyl cyclase (Sigma) and either 10 µL 4-pentyn-1-ol (for CuAAC) or 10 µL 3-azido-1-propanol (for SPAAC) for 30 min at 37°C. The reaction was stopped by adding 100 µL phenol/chloroform (5:1, pH 4.5) (Invitrogen). The RNA sample was purified with chloroform extraction and ethanol precipitated. CuAAC was performed by incubating the RNA in 100 µL reaction containing 50 mM HEPES, pH 7.0, 5 mM $MgCl_2$, 7.5 µM 16-nt RNA-azide, 1 mM $CuSO_4$, 0.5 mM THPTA and 2 mM sodium ascorbate at 25°C for 30 min. The RNA was ethanol precipitated and dissolved with RNase-free H_2O. SPAAC was performed by incubating the RNA in a 30-µL reaction containing 155.2 mM NaCl, 2.97 mM Na_2HPO_4, 1.06 mM KH_2PO_4, pH 7.4, 30 units of murine RNase inhibitor (NEB) and 25 µM 16-nt RNA-DBCO. The RNA samples were resolved on an agarose gel and stained with RedSafe (iNtRON), followed by visualization using the ChemiDoc imaging system (Bio-Rad).

Quantification of cellular ATP and NAD^+. *E. coli* K-12 MG1655 was grown in LB medium from a 1:100 dilution of a starter culture grown overnight. At a time point in log phase with an OD600 of 0.32 and a time point in stationary phase with an OD600 of 1.96, an adjusted volume of cells was removed from the culture and spun down by centrifugation at 5000 g for 4 min. Metabolites were extracted with acidic acetonitrile solvent containing acetonitrile/water (80: 20) with 0.1 M formic acid following the extraction procedure described (2). The extract was subjected to mass spectrometry under conditions described above. Concentrations were calculated using a calibration curve where concentration is plotted against chromatographic peak area.

References

1. H. Zhang et al., NAD tagSeq reveals that NAD(+)-capped RNAs are mostly produced from a large number of protein-coding genes in Arabidopsis. Proc Natl Acad Sci U S A 10.1073/pnas.1903683116 (2019).
2. J. D. Rabinowitz, E. Kimball, Acidic acetonitrile for cellular metabolome extraction from Escherichia coli. Anal Chem 79, 6167-6173 (2007).
